# Antioxidative capacity is highly associated with the storage property of tuberous roots in different sweetpotato cultivars

**DOI:** 10.1038/s41598-019-47604-8

**Published:** 2019-07-31

**Authors:** Jun Tang, Si-Qi Wang, Kang-Di Hu, Zhong-Qin Huang, Yan-Hong Li, Zhuo Han, Xiao-Yan Chen, Lan-Ying Hu, Gai-Fang Yao, Hua Zhang

**Affiliations:** 1 Xuzhou Institute of Agricultural Sciences of the Xuhuai District of Jiangsu Province, Xuzhou, Jiangsu 221131 P.R. China; 2 School of Food and Biological Engineering, Hefei University of Technology, Hefei, Anhui 230009 P.R. China; 3 Anhui Province Key Laboratory of Functional Compound Seasoning, Anhui Qiangwang seasoning Food Co., Ltd., Jieshou, 236500 P.R. China

**Keywords:** Plant breeding, Enzymes

## Abstract

The activities and gene expression of antioxidative enzymes and the ROS content were analyzed in two typical storage-tolerant cultivars (Xushu 32 and Shangshu 19) and another two storage-sensitive cultivars (Yanshu 25 and Sushu 16) to explore the association between the storage capacity of sweetpotato (*Ipomoea batatas* (L.) Lam) and ROS scavenging capability. The storage roots of the storage-tolerant cultivars maintained higher activities and expression levels of antioxidative enzymes, including ascorbate peroxidase (APX), peroxidase (POD), catalase (CAT), and superoxide dismutase (SOD); lower activity and expression of lipoxygenase (LOX); and lower accumulation of ROS metabolites compared with the storage-sensitive cultivars. The antioxidative capability and ROS parameters of leaves were positively correlated with those of storage roots. Our results provide valuable insight for evaluating the storability of sweetpotato cultivars by analyzing the capabilities of the antioxidative system and the contents of ROS metabolites.

## Introduction

Sweetpotato (*Ipomoea batatas* (L.) Lam.) is an important crop cultivated in 100 countries around the world^1^. Sweetpotatoes are rich in dietary fibers, vitamins, carotenoids, anthocyanins, flavonoids, etc.^2,3^. During storage, sweetpotato is susceptible to physiological damage, including vacuolar membrane degradation, mitochondrial membrane swelling and fungal infections^4^. A previous study showed that the optimal storage temperature for sweetpotato is 10–15 °C^5^. However, due to the lack of sophisticated facilities, enormous loss of sweetpotato yield happened in China due to chilling stress, highlighting the importance of breeding storage-tolerant sweetpotato cultivars^6^.

Postharvest storage of fruits and vegetables is accompanied by the programmed senescence of plant cells, resulting in visual and textural changes and loss in nutrient etc. Many environmental and internal factors including storage temperature, humidity, and phytohormones affect postharvest senescence and decay^7–10^. Among the stress signals, reactive oxygen species (ROS), particularly H_2_O_2_ and O_2_^−^ accumulation are closely related to plant senescence^11^. Oxidative damages caused by excessive ROS result in mitochondria dysfunction, enzyme inactivation and lipid peroxidation^12^. Thus ROS detoxification is crucial for the balance of ROS accumulation. Plants have evolved non-enzymatic and enzymatic antioxidant systems to scavenge excessive ROS, and the enzymatic antioxidant system includes multiple components, such as ascorbate peroxidase (APX), superoxide dismutase (SOD), catalase (CAT) and peroxidase (POD)^11,13^. Accumulating studies demonstrated that the enhancement of cellular antioxidant system could delay senescence by removing excess ROS in different plants^14,15^. Therefore, an increased ROS scavenging capacity is positively correlated with the prevention of postharvest senescence.

Although previous studies reported progresses in optimizing the storage conditions during postharvest sweetpotato storage, what endogenous factors contribute to the storage properties of different cultivars are still unclear^4,6,16^. As ROS is an elicitor of postharvest senescence, we hypothesized that increased antioxidative enzymes could be positively associated with the storage property of sweetpotatoes. In our recent report, low-temperature storage was found to induce ROS accumulation and antioxidant enzymes were rapidly enhanced by chilling stress^17^. However, whether the ability of ROS scavenging is associated with storage ability is still unclear. Besides, the antioxidative capability in leaves might be positively correlated with those in tuberous roots, but this hypothesis still needs more investigation. In this research, a sweetpotato cultivar with a rot rate of less than 75% after 290 days of storage at 11–15 °C was classified as a storage-tolerant cultivar. A lower rot rate indicates a higher storage property. A sweetpotato cultivar with a rot rate of more than 75% was classified in the storage-sensitive varieties. The activities and gene expression of antioxidative enzymes and the ROS contents were investigated in the storage roots and leaves of two typical storage-tolerant cultivars (Xushu 32 and Shangshu 19) and two storage-sensitive cultivars (Yanshu 25 and Sushu 16), thereby exploring the relationship between storage behavior and antioxidative capability in sweetpotato. Furthermore, the correlation and principal component analyses were processed to reveal the correlation between antioxidative parameters and the clustering of sweetpotato cultivars, respectively.

## Results

### Enzymatic activities of APX, POD, CAT, SOD, PPO and LOX in the storage roots of sweetpotato cultivars

**Figure 1:**
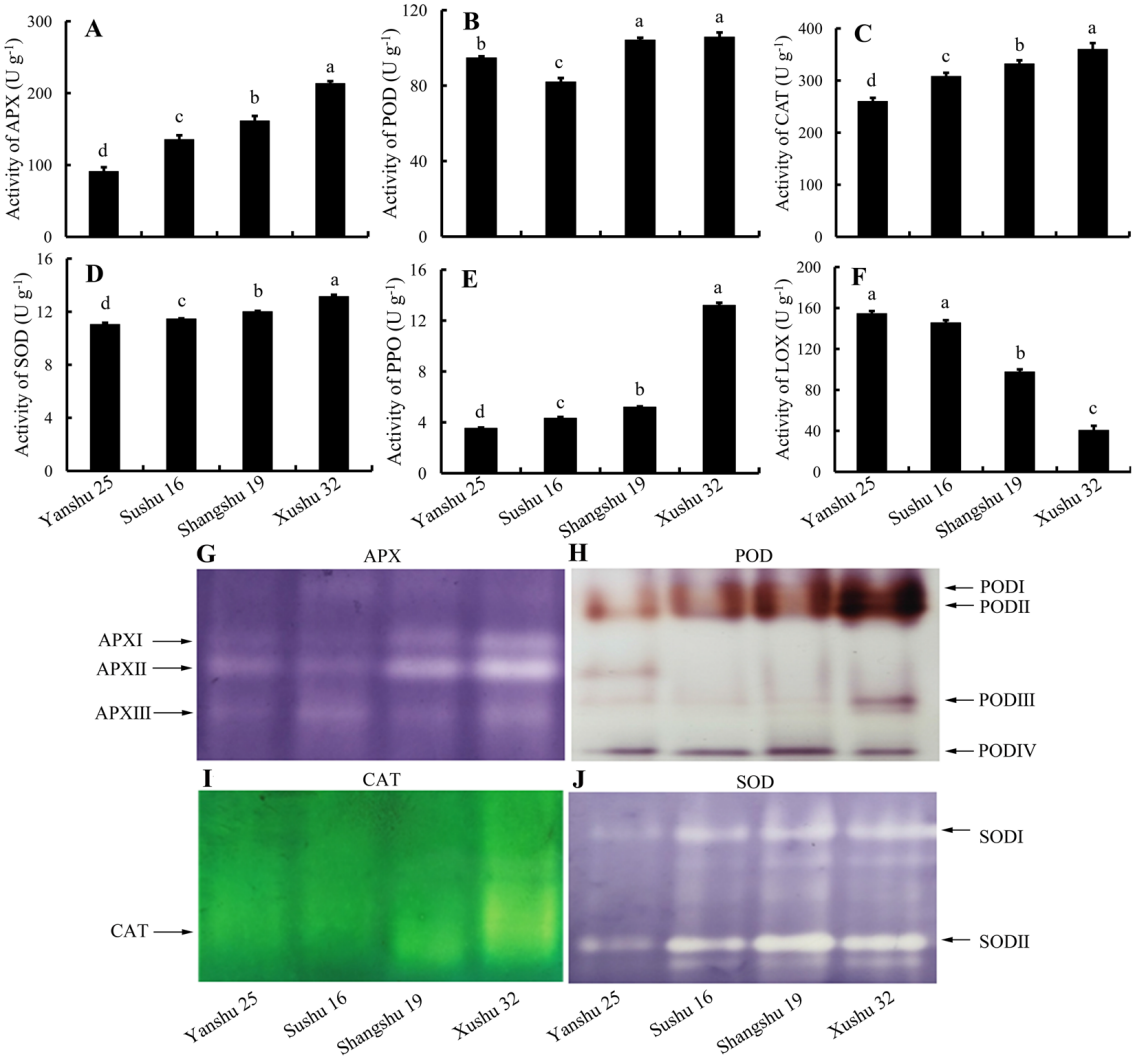
Activities of ascorbate peroxidase (APX) (**A**), peroxidase (POD) (**B**), catalase (CAT) (**C**), superoxide dismutase (SOD) (**D**), polyphenol oxidase (PPO) (**E**) and lipoxygenase (LOX) (**F**) in tuberous roots of the sweetpotato cultivars Yanshu 25, Sushu 16, Shangshu 19 and Xushu 32. Data are presented as the means ± SD (n = 3). Different letters indicate significant differences (p < 0.05) according to t-tests. Native polyacrylamide gel electrophoresis (PAGE) of the isozyme profile of ascorbate peroxidase (APX) (**G**), peroxidase (POD) (**H**), catalase (CAT) (**I**), and superoxide dismutase (SOD) (**J**) in the tuberous roots of sweetpotato cultivars Yanshu 25, Sushu 16, Shangshu 19 and Xushu 32.

The sweetpotato cultivars Yanshu 25 and Sushu 16 with lower storage properties and two typical storage-tolerant cultivars, Shangshu 19 and Xushu 32, were selected to study the possible relationships between the ROS scavenging capability and the storage properties of sweetpotato. The activities of the antioxidative enzymes and lipid peroxidation-related enzyme, LOX, were analyzed in the storage roots of the four sweetpotato cultivars. As shown in Fig. 1A, APX activities in the storage-tolerant cultivars Xushu 32 and Shangshu 19 were maintained at a higher level than those in Yanshu 25 and Sushu 16. In addition, a lower level of APX activity was observed in Shangshu 19 compared with Xushu 32 and in Yanshu 25 compared with Sushu 16. POD activity in sweetpotato roots is shown in Fig. 1B. Yanshu 25 and Sushu 16 maintained lower POD activities compared with Xushu 32 and Shangshu 19, but a 13% higher POD activity appeared in Yanshu 25 compared to Sushu 16. Figure 1C–E shows similar results in the activities of CAT, SOD and PPO in sweetpotato roots. The activities of CAT, SOD and PPO in the storage-tolerant cultivars Xushu 32 and Shangshu 19 were always higher than those of Yanshu 25 and Sushu 16. CAT, SOD and PPO activities in Xushu 32 were higher than those in Shangshu 19. Those in Sushu 16 were higher than those in Yanshu 25. LOXs belong to a large family of plant enzymes that catalyze the hydroperoxidation of polyunsaturated fatty acids^18^. Figure 1F shows that the storage-tolerant cultivars Shangshu 19 and Xushu 32 sustained a lower level of LOX compared to Yanshu 25 and Sushu 16. Xushu 32 showed only approximately half the LOX activity shown by Shangshu 19. These results suggest that the activities of antioxidative enzymes in storage roots were maintained at higher levels in the storage-tolerant cultivars Xushu 32 and Shangshu 19 in comparison to the storage-sensitive cultivars Yanshu 25 and Sushu 16. Similarly, LOX activity was opposite to the pattern of antioxidant enzymes in the four sweetpotato cultivars.

### Isoenzyme analysis of APX, POD, CAT and SOD in the storage roots of sweetpotato

To gain insight into the isoenzyme changes in the antioxidant enzymes in sweetpotato roots, the activities of the antioxidant enzymes were investigated by native PAGE. Figure 1G–J shows that the reaction bands of APX, POD, CAT and SOD in the storage roots of storage-tolerant cultivars Xushu 32 and Shangshu 19 were significantly brighter (more intense) than those of the storage-sensitive cultivars Yanshu 25 and Sushu 16, suggesting that higher antioxidative enzyme activities exist in the storage roots of Xushu 32 and Shangshu 19 compared with Yanshu 25 and Sushu 16. As shown in Fig. 1G, the APX isoforms APX I and APX II showed increased activity in the two storage-tolerant cultivars Xushu 32 and Shangshu 19 compared with the other two cultivars. POD I and POD II showed higher band intensities in the roots of the two storage-tolerant cultivars (Fig. 1H). An increase in POD III was observed in the most storage-tolerant cultivar, Xushu 32. Additionally, Xushu 32 and Shangshu 19 showed higher CAT activities compared with the two storage-sensitive cultivars (Fig. 1I). SOD I and SOD II displayed increased activity in storage-tolerant Xushu 32 and Shangshu 19 in comparison to the storage-sensitive cultivars (Fig. 1J). Furthermore, the activities of SOD I and SOD II were higher in Sushu 16 than in the most storage-sensitive cultivar, Yanshu 25. Thus, the results of the native PAGE were consistent with the spectrophotometric activity analysis of the antioxidative enzymes.

### Contents of hydrogen peroxide and malondialdehyde and the production of superoxide anion in sweetpotato roots

**Figure 2:**
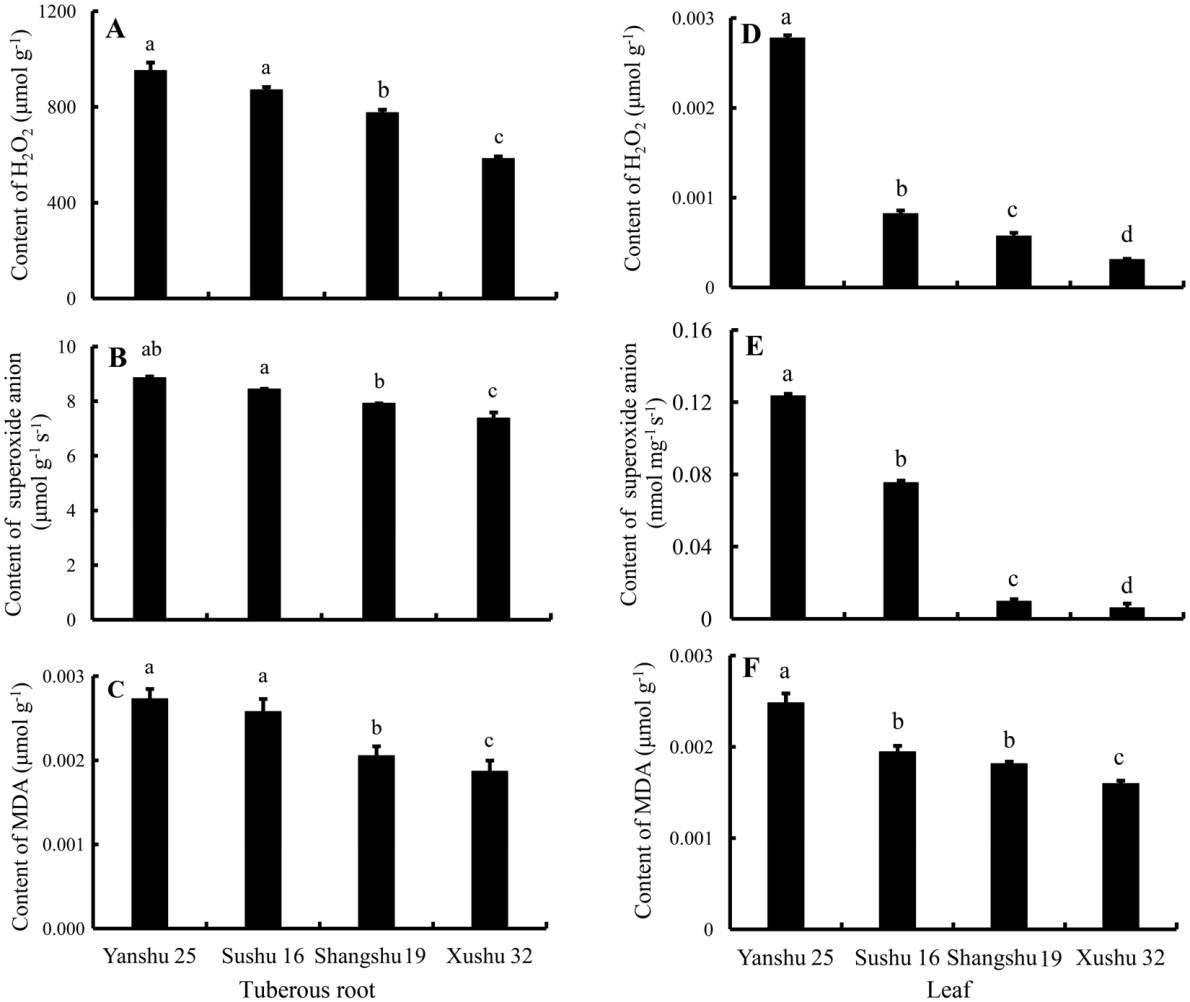
Hydrogen peroxide (H_2_O_2_) content (**A**,**D**), production of superoxide anions (⋅O_2_^−^) (**B**,**E**) and content of malondialdehyde (MDA) (**C**,**F**) in the tuberous roots and leaves of sweetpotato cultivars Yanshu 25, Sushu 16, Shangshu 19 and Xushu 32. Data are presented as the means ± SD (n = 3). Different letters indicate significant differences (p < 0.05) according to t-test.

Accumulated ROS induce oxidative damage and are implicated in the postharvest senescence process, and MDA is an index of lipid peroxidation^12,19,20^. Thus, the contents of H_2_O_2_ and MDA and the production of ⋅O_2_^−^ in sweetpotato roots were analyzed and shown in Fig. 2A–C. As shown in Fig. 2A, the storage-tolerant cultivars Xushu 32 and Shangshu 19 maintained lower H_2_O_2_ contents compared with Yanshu 25 and Sushu 16, and Xushu 32 had the lowest level. Figure 2B,C illustrate similar patterns for ⋅O_2_^−^ production and MDA content as for the H_2_O_2_ content. The generation of ⋅O_2_^−^ and MDA in storage-sensitive Yanshu 25 and Sushu 16 was significantly higher compared with Xushu 32 and Shangshu 19. In addition, the most storage-tolerant cultivar, Xushu 32, also contained the lowest level of ROS metabolites compared with the other three cultivars.

### Antioxidative enzyme activities and ROS metabolites analysis in sweetpotato leaves

**Figure 3:**
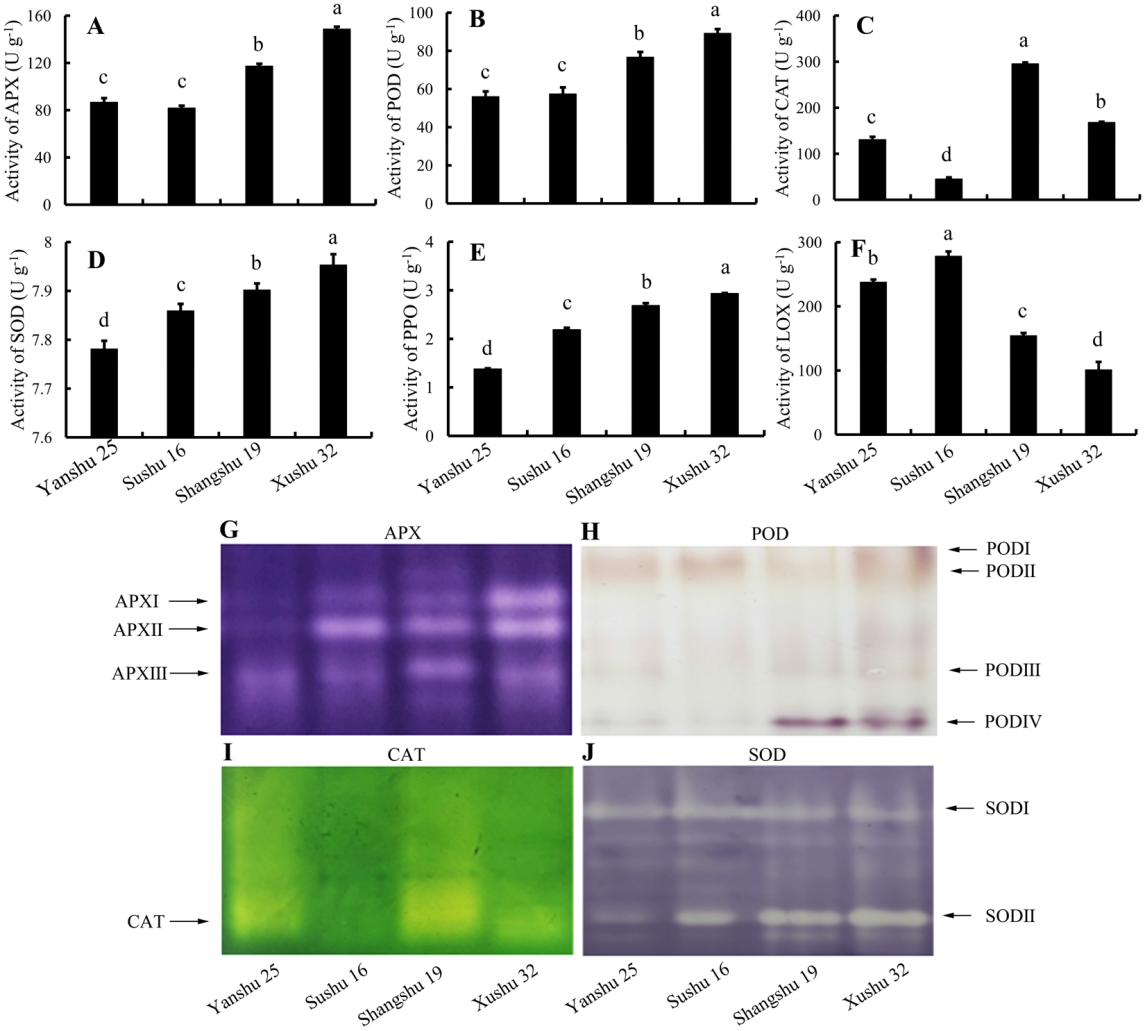
Activities of ascorbate peroxidase (APX) (**A**), peroxidase (POD) (**B**), catalase (CAT) (**C**), superoxide dismutase (SOD) (**D**), polyphenol oxidase (PPO) (**E**) and lipoxygenase (LOX) (**F**) in leaves of sweetpotato cultivars Yanshu 25, Sushu 16, Shangshu 19 and Xushu 32. Data are presented as the means ± SD (n = 3). Different letters indicate significant differences (p < 0.05) according to t-tests. Native polyacrylamide gel electrophoresis (PAGE) of the isozyme profile of ascorbate peroxidase (APX) (**G**), peroxidase (POD) (**H**), catalase (CAT) (**I**), and superoxide dismutase (SOD) (**J**) in the leaves of sweetpotato cultivars Yanshu 25, Sushu 16, Shangshu 19 and Xushu 32.

**Figure 4:**
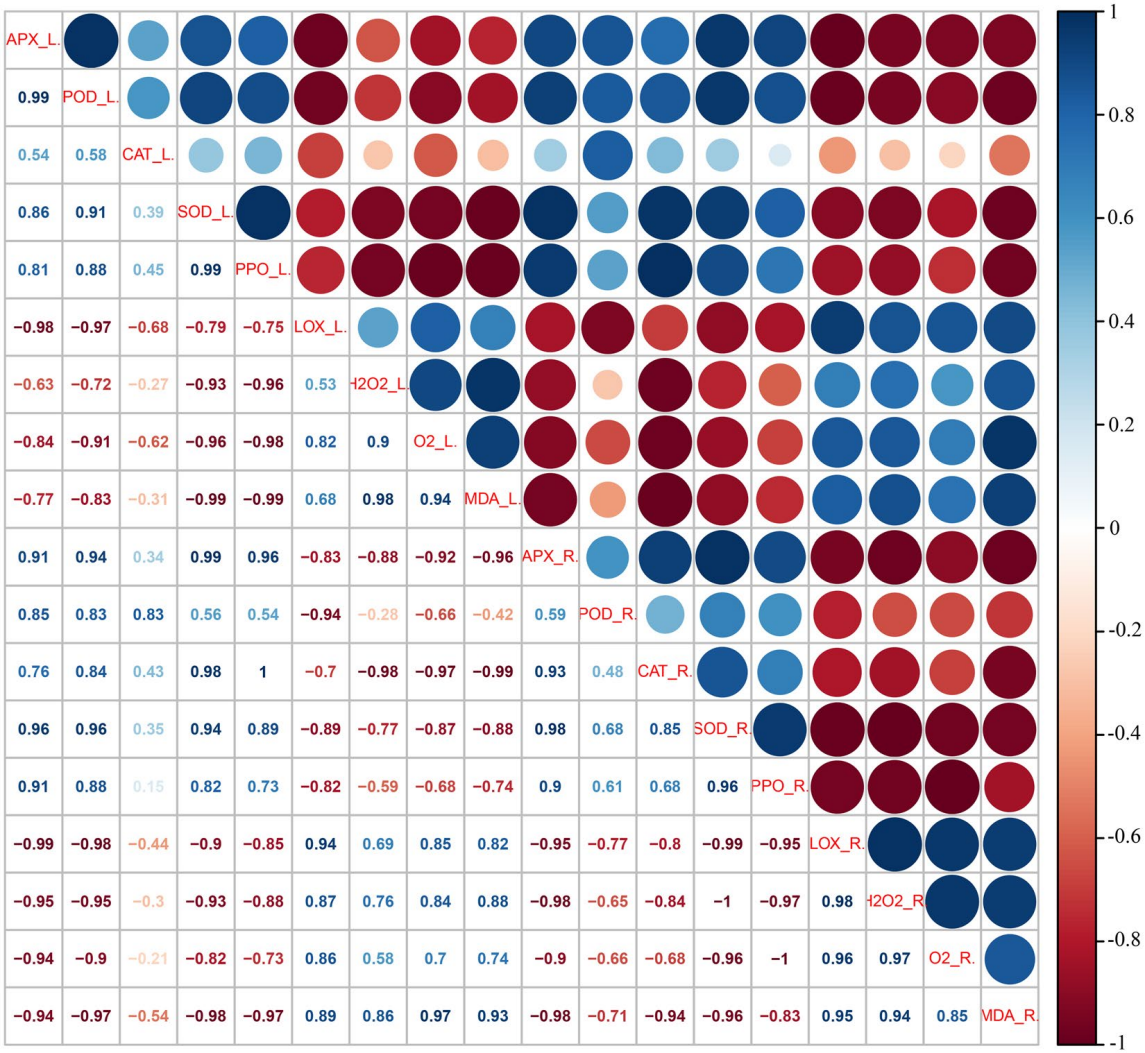
Correlation analysis among the parameters of ascorbate peroxidase (APX), peroxidase (POD), superoxide dismutase (SOD), catalase (CAT) and lipoxygenase (LOX) and the data of hydrogen peroxide (H_2_O_2_), production of superoxide anion (⋅O_2_^−^) and content of malondialdehyde (MDA) in tuberous roots and leaves of sweetpotato cultivars Yanshu 25, Sushu 16, Shangshu 19 and Xushu 32. Pearson’s correlation coefficient among data was analyzed using R scripts. R, the abbreviation of root; L, the abbreviation of leave.

To explore whether similar ROS metabolism existed in sweetpotato leaves as in storage roots, antioxidative enzyme activities and ROS metabolites were analyzed in sweetpotato leaves. As shown in Fig. 3A–E, the activities of APX, POD, CAT, SOD and PPO in the leaves of the storage-tolerant cultivars Xushu 32 and Shangshu 19 were always higher than those of storage-sensitive Yanshu 25 and Sushu 16. In addition, the activities in the native PAGE showed consistent results with the activity determination data (Fig. 3G–J). Figure 3F shows that the storage-tolerant cultivars Shangshu 19 and Xushu 32 sustained a significantly lower level of LOX compared with Yanshu 25 and Sushu 16. Figure 2D–F showed that the ROS metabolites and MDA content in the leaves of the storage-sensitive cultivars Yanshu 25 and Sushu 16 were significantly higher than those in the two storage-tolerant cultivars Shangshu 19 and Xushu 32, which were similar to the results in sweetpotato roots except for the insignificant MDA values between Sushu 16 and Shangshu 19. In addition, Pearson correlation analysis was performed among the antioxidative enzyme activities and ROS metabolites in the roots and leaves of the four sweetpotato cultivars (Fig. 4). A perusal of the data revealed that the activities of antioxidative enzymes and ROS metabolites in the sweetpotato roots had a highly significant and positive correlation with the corresponding parameters in the leaves, such as APX activity (*r* = 0.910), POD activity (*r* = 0.834), CAT activity (*r* = 0.430), SOD activity (*r* = 0.940), LOX activity (*r* = 0.943), H_2_O_2_ content (*r* = 0.758), ⋅O_2_^−^ production (*r* = 0.912) and MDA content (*r* = 0.934), suggesting that similar ROS scavenging capacity and ROS metabolites existed in sweetpotato leaves and roots. In addition, the data of ROS metabolites, H_2_O_2_, ⋅O_2_^−^ and MDA were negatively correlated with the activities of ROS scavenging enzymes and positively correlated with LOX.

### Relative gene expression of antioxidative enzyme genes and LOX in sweetpotato roots and leaves

**Figure 5:**
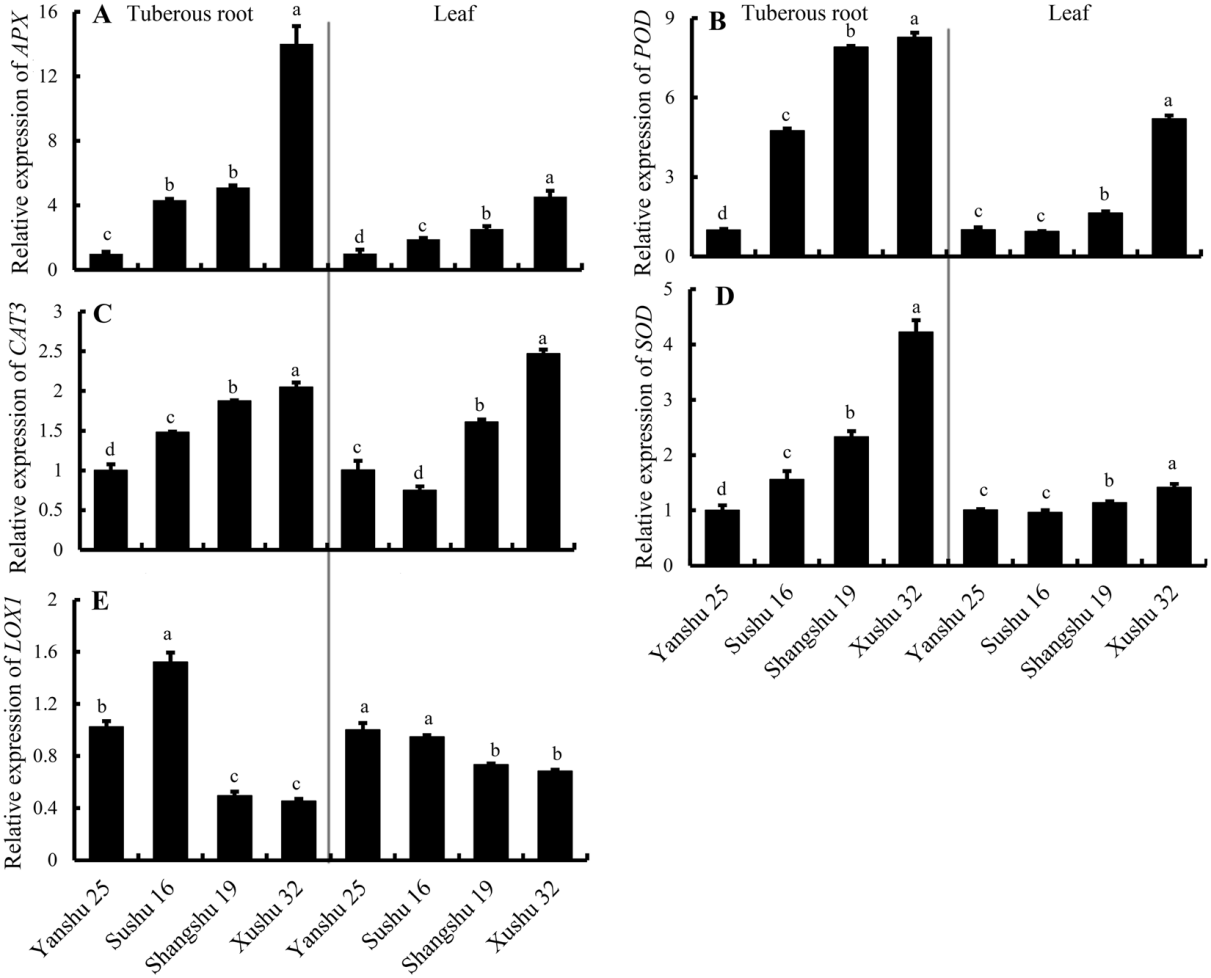
Relative gene expression levels of itf09g09790 (*IbAPX*) (**A**), itf09g09800 (*IbPOD*) (**B**), itf07g00160 (*IbCAT3*) (**C**), itf13g19030 (*IbSOD*) (**D**), itf15g12180 (*IbLOX1*) (**E**) in the tuberous roots and leaves of sweetpotato cultivars Yanshu 25, Sushu 16, Shangshu 19 and Xushu 32.

**Figure 6:**
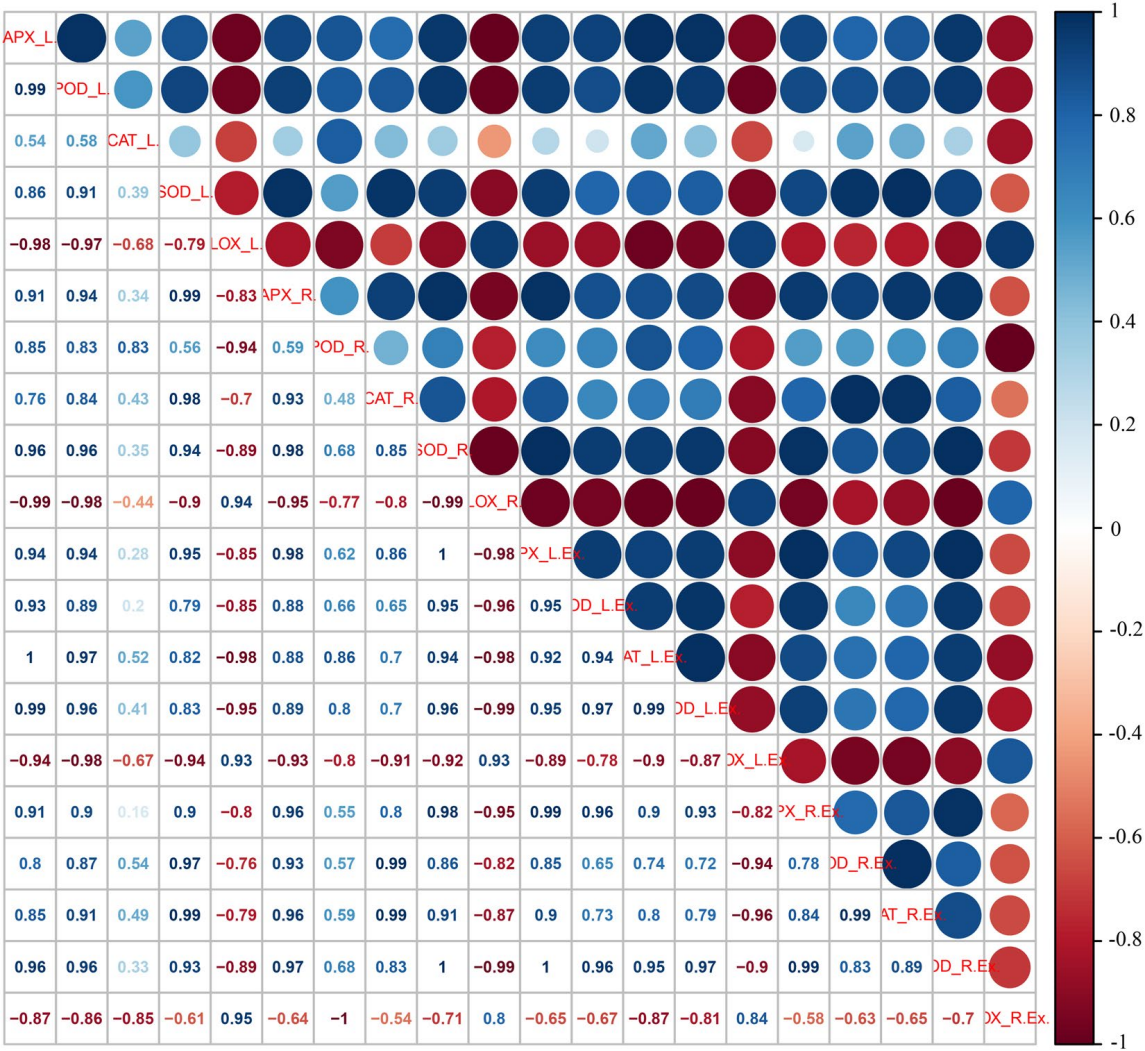
Correlation analysis among the parameters of ascorbate peroxidase (APX), peroxidase (POD), superoxide dismutase (SOD), catalase (CAT) and lipoxygenase (LOX) and gene expression of itf09g09790 (*IbAPX*), itf13g19030 (*IbSOD*), itf09g09800 (*IbPOD*), itf07g00160 (*IbCAT3*), itf15g12180 (*IbLOX1*) in tuberous roots and leaves of sweetpotato cultivars Yanshu 25, Sushu 16, Shangshu 19 and Xushu 32. Pearson’s correlation coefficient among data was analyzed using R scripts. R, abbreviation of root; L, abbreviation of leave; Ex, abbreviation of gene expression.

To investigate the transcription levels of the genes encoding antioxidative enzymes in sweetpotato, their relative gene expression in sweetpotato roots and leaves was assayed by quantitative PCR. As shown in Fig. 5, the expression of *IbAPX* in the roots of storage-tolerant Xushu 32 and Shangshu 19 was significantly higher compared with Yanshu 25 and Sushu 16 and was nearly 4-fold higher than Yanshu 25. *IbSOD*, *IbPOD* and *IbCAT3* in sweetpotato roots showed similar gene expression patterns among the four cultivars to the data of *IbAPX* in roots. However, the *IbLOX1* gene was expressed at higher levels in the storage roots of storage-sensitive Yanshu 25 and Sushu 16 than in the other two storage-tolerant cultivars. In addition, the relative gene expression in sweetpotato leaves showed a similar expression pattern to the root data. Generally, the genes encoding ROS scavenging enzymes were expressed at higher levels in the storage-tolerant cultivars, whereas *IbLOX1* expression was lower in comparison to the data in the leaves of the storage-sensitive varieties. Meanwhile, the correlation among the antioxidative enzyme activities and the corresponding gene expression in sweetpotato roots and leaves were analyzed and are presented in Fig. 6. The data show that the activities of antioxidant enzymes in sweetpotato roots and leaves had highly significant and positive correlations with their corresponding genes, and the correlation coefficients ranged from 0.517 to 0.958. In addition, the gene expression in sweetpotato roots had a highly significant and positive correlation with the corresponding genes in the leaves, such as *IbAPX* (*r* = 0.991), *IbSOD* (*r* = 0.969), *IbPOD* (*r* = 0.647), *IbCAT3* (*r* = 0.798) and *IbLOX1* (*r* = 0.845), suggesting that similar gene expression exists in the roots and leaves of sweetpotato.

### Antioxidative enzymes and related gene expression analysis in four other sweetpotato cultivars

To verify the association between higher antioxidative capability and better storage properties, four other sweetpotato cultivars with different storabilities, Jishu 26, Guangshu 87, Zhezi 3 and Yushu, were selected. As shown in Fig. S1, the results of native PAGE of the antioxidative enzymes in the roots and leaves showed that the storage-tolerant cultivars Jishu 26 and Guangshu 87 maintained higher APX and CAT activities compared with the storage-sensitive cultivars Zhezi 3 and Yushu, which is similar to the activity assay of APX and CAT in Fig. S2. The storage-tolerant cultivars Jishu 26 and Guangshu 87 maintained higher *IbAPX* gene expression. The storage-sensitive cultivars Zhezi 3 and Yushu showed higher LOX activity and higher H_2_O_2_ content compared with the storage-tolerant cultivars Jishu 26 and Guangshu 87. Meanwhile, correlations among APX, CAT, and LOX activities and the content of H_2_O_2_ and APX gene expression in the four sweetpotato cultivars of were analyzed and are presented in Fig. S3. The correlation data showed that the activities of APX, CAT, and LOX and the content of H_2_O_2_ and APX gene expression had a positive correlation in sweetpotato roots and leaves, and the correlation coefficients ranged from 0.67 to 0.99. The principal component (PC) analysis in Fig. S4 shows that PC 1 and 2 explained 79.898% and 16.357% of the variability in the data. There is a clear separation between the storage-tolerant cultivars and storage-sensitive cultivars in PC1 according to the above measured parameters. In addition, the cultivar displaying the highest positive loading value in the direction of PC1 was Xushu 32, and Sushu 16 on PC2 exhibited the lowest negative loading score, suggesting a correlation with storage. Thus, the positive association between antioxidative capabilities and storage properties might be universal in sweetpotato cultivars with different storabilities.

## Discussion

Senescence greatly impacts fruit or crop postharvest quality and resistance to pathogen attack^12^. Oxidative damage caused by ROS is one of the most important factors that cause plant senescence^14^. The induction of cellular antioxidant machinery has been applied to protect plants from oxidative stress and to alleviate postharvest senescence^21,22^. Thus, we propose the hypothesis that the innate ROS scavenging capability is positively associated with the storage property of sweetpotatoes. Studies have shown that chilling stress, disease and lack of oxygen strongly affect the storage of sweetpotatoes^23^. Sweetpotatoes stored in developing countries is more susceptible to chilling damage due to equipment problems^16^. Considering the future demand for food, species of sweetpotato that are resistant to low temperatures, salinity, and water stress will expand the crop area and production^24^. Therefore, it is necessary to explore the relationship between the storage of sweetpotato and its antioxidant capacity.

Storage-tolerant cultivars Xushu 32 and Shangshu 19 maintained higher antioxidant enzyme activities for APX, POD, CAT, and SOD and lower LOX activity in comparison to the storage-sensitive cultivars Yanshu 25 and Sushu 16 in sweetpotato roots, as shown in Fig. 1. Lower contents of ROS metabolites, including H_2_O_2_, ⋅O_2_^−^ and MDA, are observed in the roots of storable cultivars, Xushu 32 and Shangshu 19, in comparison to Yanshu 25 and Sushu 16. LOXs are a large family of plant enzymes that catalyze the hydroperoxidation of polyunsaturated fatty acids and lead to the production of MDA. The above results suggest that antioxidative enzyme activities have a significant positive correlation with the storability of sweetpotato, while the contents of ROS metabolites and LOX activity show a negative correlation. Our results are consistent with data showing that storage-tolerant soybeans maintain a higher level of antioxidant enzyme activities^25^. Accumulation of ROS can cause plant tissue damage and reduce the storage quality of fruits and vegetables^19,26^. In sweetpotato, transcriptome profiling of storage roots during low-temperature storage shows that the gene expression of SOD and CAT is downregulated with a concurrent increase in H_2_O_2_ and MDA, emphasizing the key role of the ROS scavenging capability in sweetpotato storage^16^. Plants have evolved an efficient antioxidant system that includes enzymes such as SOD, CAT, POD and APX to scavenge ROS to avoid oxidative damage caused by accumulated ROS^27^. Among them, two major antioxidant enzymes in plants provide the primary defense against ROS: SOD catalyzes the conversion of ⋅O_2_^−^ into H_2_O_2_, and CAT removes the resultant H_2_O_2_. APX and POD are the key enzymes responsible for H_2_O_2_ scavenging during oxidative stress in plants. However, oxidative stress may still occur during postharvest storage due to the gradual loss of ROS scavenging enzymes despite such an efficient defense system^28^. Studies have revealed that tomato and guava cultivars with longer shelf lives exhibit higher activities of ROS scavenging enzymes and thus experience less oxidative stress^28,29^. Thus, an efficient antioxidative system at the beginning of storage can protect postharvest crops or fruits from the deleterious effects of ROS. Antioxidative capability is positively correlated with storage in sweetpotato.

A similar pattern of antioxidant enzyme activities and ROS metabolite contents is observed in sweetpotato leaves as in storage roots, as shown in Figs 2 and 3. Correlation among the data in sweetpotato roots and leaves (Fig. 4) also suggests that the activities of antioxidative enzymes and ROS metabolites in sweetpotato roots have a positive correlation with the corresponding parameters in leaves. The positive correlation of data between roots and leaves suggests that the antioxidative capability determined in leaves can be associated with the storage properties of storage roots. Consistent with the results of the enzyme activity assay, the antioxidant enzyme genes expression *IbAPX*, *IbSOD*, *IbPOD* and *IbCAT3* were expressed at higher levels in the roots and leaves of the storage-tolerant cultivars Xushu 32 and Shangshu 19 than in the two storage-sensitive cultivars, whereas *IbLOX1* showed the opposite expression pattern (Fig. 5). There is a highly positive correlation between antioxidative enzyme activities and corresponding gene expression in sweetpotato (Fig. 6).

To examine associations between higher antioxidative capabilities and better storage properties, some parameters were analyzed in four other sweetpotato cultivars with different storabilities: Jishu 26, Guangshu 87, Zhezi 3 and Yushu. Storage-tolerant Jishu 26 and Guangshu 87 maintained higher APX and CAT activities and higher gene expression compared with the storage-sensitive cultivars Zhezi 3 and Yushu in both roots and leaves, while the storage-sensitive cultivars Zhezi 3 and Yushu contained higher LOX activity and H_2_O_2_ content. Thus, the positive association between antioxidative capabilities and storage properties might be universal in sweetpotato cultivars.

In conclusion, our results indicate that the storage-tolerant sweetpotato cultivars maintained higher antioxidant enzyme activities and gene expression and lower levels of ROS metabolites compared with the storage-sensitive cultivars. Meanwhile, the data for antioxidative enzyme activities, gene expression and ROS metabolites in storage roots showed a correlation with those in leaves, which provides valuable markers for the breeding of sweetpotatoes. The significant positive correlation between enzyme activities and corresponding gene expression suggests that the transcription levels of antioxidant genes could reflect antioxidative capacities in leaves and storage roots. Thus, this new strategy of storability evaluation will facilitate and shorten the breeding cycle of sweetpotato varieties with higher storage properties.

## Materials and Methods

### Plant materials and sample preparation

The sweetpotato stem cuttings (*I*. *batatas* cv. Xushu 32, Shangshu 19, Yanshu 25, Sushu 16, Yushu, Zhezi 3, Guangshu 87, and Jishu 26) were carried out in May 2017 at the National Sweet Potato Improvement Center, Xuzhou, Jiangsu Province, China. The second-to-last through the fifth-to-last leaves of each sweetpotato cultivar were sampled immediately upon arrival. The leaves of each cultivar were pre-cooled with liquid nitrogen, mixed, ground, and then stored in a −80 °C freezer until experimental assay. The corresponding storage roots of the eight cultivars were harvested in October 2017 from the National Sweet Potato Improvement Center. Five unblemished and disease-free storage roots were selected for each cultivar. Five roots of individual cultivars were cut into small pieces, and 100 g of each root was sampled and mixed to eliminate the effects of individual variance, immediately frozen in liquid nitrogen and stored in the −80 °C freezer. The cultivars with a rot rate of less than 75% after 290 days of sweetpotato root storage at 11−15 °C were defined as a storage-tolerant species. A lower rot rate indicates a higher storage property. A plant with a rot rate of more than 75% was defined as a storage-sensitive cultivar. Eight sweetpotato varieties were stored at 11–15 °C at 80–90% humidity for 290 days in an air-conditioned room, and their storage properties were determined via the rot rate of the roots. After storage for 290 days, Yanshu 25 was fully rotten, while the rot rate of Yushu was 95%, Zhezi 3 was 80%, Sushu 16 was 75%, Guangshu 87 was 72%, Shangshu 19 was 70%, Jishu was 67%, and Xushu 32 was 65%. The storage property was also studied of the sweet potato cultivars in the year of 2015 and 2016 and similar results were obtained. All samples were prepared in three biological replicates taken from the mixed leaves and the same five pooled roots.

### Activity assay of antioxidative enzymes and lipoxygenase

Ascorbate peroxidase (EC 1.11.1.11), peroxidase (EC 1.11.1.7), catalase (EC 1.11.1.6) and superoxide dismutase (EC 1.15.1.1) activities were determined according to the method of García-Limones *et al*.^30^. The enzymes in sweetpotato leaves and storage roots were sampled and extracted according to the method in Wang *et al*.^17^. The content of soluble protein in the samples was determined by the method described by Bradford^31^. The activity was expressed on a protein basis as U·g^−1^.

The activities of LOX (EC 1.13.11.12) were determined by the procedures described by Surrey^32^. One unit of LOX was defined as a decrease of 0.01 OD value in absorbance per minute, and the results were expressed on a protein basis as U·g^−1^.

### Electrophoretic analysis of APX, POD, CAT and SOD

The APX, POD, CAT and SOD isoenzymes were separated by native polyacrylamide gel electrophoresis (PAGE)^33^ using a Mini-Protein II electrophoresis system (Bio-Rad Laboratories, CA). Equal amounts of protein (16 μg) were loaded and under electrophoresis for 4 h using a 25 mA current.

The isozymes of APX were stained according to the method described by Mittler and Zilinskas^34^, which is based on the inhibition of NBT reduction by ascorbate. The isozymes of POD were detected based on the method of Guikema and Sherman^35^. CAT activity was shown according to the descriptions of Clare, *et al*.^36^. The SOD activity were stained following the method of Beauchamp and Fridovich^37^.

### Determination of the contents of hydrogen peroxide and malondialdehyde and production of superoxide anions in sweetpotato tubers and leaves

The contents of H_2_O_2_ and malondialdehyde (MDA) and the production of ⋅O_2_^−^ were assayed as described by Ge *et al*.^38^ and Hu *et al*.^19^. The ROS metabolites in sweetpotato leaves and storage roots were sampled, extracted and calculated according to the method in Wang *et al*.^17^.

### Quantitative reverse transcription PCR analysis

Sweetpotato leaves and storage root samples (0.2 g) were ground in liquid nitrogen, and the total RNA was extracted using a TRNzol RNA Reagent kit (Tiangen, Beijing, China) following the manufacturer’s instructions and used for cDNA synthesis by a reverse transcription kit (PrimeScript RT Master Mix, Takara, Kyoto, Japan). Quantitative PCR was carried out in three replicates using an iQTM5 PCR System with SYBR Premix Ex Taq (Takara, Kyoto, Japan). The following genes itf09g09790 (*IbAPX*), itf13g19030 (*IbSOD*), itf09g09800 (*IbPOD*), itf07g00160 (*IbCAT3*), and itf15g12180 (*IbLOX1*) and the housekeeping gene itf04g29110 (*IbTubulin*, reference gene) were obtained following the method in Wang *et al*.^17^. The primers used for quantitative PCR are shown in Table S1.

### Statistical analysis

Statistical significance was analyzed with *t*-tests that were conducted using IBM SPSS Statistics (SPSS version 22.0; Armonk, NY), and the results were expressed as the means ± SD (standard deviation). Native PAGE analysis of APX, CAT, POD and SOD was repeated three times, and similar results were obtained. The Pearson correlation coefficient (R) was used to show the correlation among enzyme activities, gene expression, and ROS metabolites in the storage roots and leaves of the sweetpotato cultivars. Principal component (PC) analysis was performed using IBM SPSS Statistics.

## Supplementary information

Dataset 1

## Data Availability

All materials, data and associated protocols are available upon request.

## References

[1] Pradhan DMP (2015). High starch, beta carotene and anthocyanin rich sweet potato: ascent to future food and nutrition security in coastal and backward areas. Int. J. Trop. Agric..

[2] Kang L (2017). Suppression of the *β-carotene hydroxylase* gene increases β-carotene content and tolerance to abiotic stress in transgenic sweetpotato plants. Plant Physiol. Biochem..

[3] Wang S, Nie S, Zhu F (2016). Chemical constituents and health effects of sweet potato. Food Res. Int..

[4] Padda MS, Picha DH (2008). Effect of low temperature storage on phenolic composition and antioxidant activity of sweetpotatoes. Postharvest Biol. Technol..

[5] van Oirschot Q, Rees D, Aked J, Kihurani A (2006). Sweetpotato cultivars differ in efficiency of wound healing. Postharvest Biol. Technol..

[6] Xie Z (2017). High throughput deep sequencing reveals the important roles of microRNAs during sweetpotato storage at chilling temperature. Sci. Rep..

[7] Adams-Phillips L (2004). Evidence that CTR1-Mediated ethylene signal transduction in tomato is encoded by a multigene family whose members display distinct regulatory features. Plant Mol. Biol..

[8] Giovannoni JJ (2004). Genetic regulation of fruit development and ripening. Plant Cell.

[9] Klee HJ, Giovannoni JJ (2011). Genetics and control of tomato fruit ripening and quality attributes. Annu.Rev. Genet..

[10] Qin G (2012). Unraveling the regulatory network of the MADS box transcription factor RIN in fruit ripening. Plant J..

[11] Raseetha S (2013). Evolution of antioxidant enzymes activity and volatile release during storage of processed broccoli (*Brassica oleracea* L. *italica*). LWT-Food Sci. Tech..

[12] Tian S, Qin G, Li B (2013). Reactive oxygen species involved in regulating fruit senescence and fungal pathogenicity. Plant Mol. Biol..

[13] Mittler R, Vanderauwera S, Gollery M, Van Breusegem F (2004). Reactive oxygen gene network of plants. Trends Plant Sci..

[14] Qin G, Meng X, Wang Q, Tian S (2009). Oxidative damage of mitochondrial proteins contributes to fruit senescence: a redox proteomics analysis. J. Proteome Res..

[15] Zimmermann P, Heinlein C, Orendi G, Zentgraf U (2006). Senescence-specific regulation of catalases in *Arabidopsis thaliana* (L.) Heynh. Plant Cell Environ..

[16] Ji CY (2017). Transcriptome profiling of sweetpotato tuberous roots during low temperature storage. Plant Physiol. Biochem..

[17] Wang S (2019). Antioxidative System in Sweet Potato Root is Activated by Low Temperature Storage. J. Sci. Food Agric..

[18] Hansen J (2013). Bacterial lipoxygenases, a new subfamily of enzymes? A phylogenetic approach. Appl. Microbiol. Biotechnol..

[19] Hu L (2012). Hydrogen sulfide prolongs postharvest shelf life of strawberry and plays an antioxidative role in fruits. J. Agric. Food Chem..

[20] Li S (2014). Hydrogen sulfide alleviates postharvest senescence of broccoli by modulating antioxidant defense and senescence-related gene expression. J. Agric. Food Chem..

[21] Yan H (2016). Overexpression of *CuZnSOD* and *APX* enhance salt stress tolerance in sweet potato. Plant Physiol. Biochem..

[22] Tuteja N (2007). Mechanisms of high salinity tolerance in plants. Methods Enzymol..

[23] Fan W (2015). Elevated compartmentalization of Na^+^ into vacuoles improves salt and cold stress tolerance in sweet potato (*Ipomoea batatas*). Physiol. Plant..

[24] Fan W, Zhang M, Zhang H, Zhang P (2012). Improved tolerance to various abiotic stresses in transgenic sweet potato (*Ipomoea batatas*) expressing spinach betaine aldehyde dehydrogenase. PLoS One..

[25] Hosamani J (2013). Biochemical phenotyping of soybean [*Glycine max* (L.) Merill] genotypes to establish the role of lipid peroxidation and antioxidant enzymes in seed longevity. Agric. Res..

[26] Hodges DM, Lester GE, Munro KD, Toivonen PMA (2004). Oxidative stress: importance for postharvest quality. HortScience..

[27] Gill SS, Tuteja N (2010). Reactive oxygen species and antioxidant machinery in abiotic stress tolerance in crop plants. Plant Physiol. Biochem..

[28] Mondal K (2004). Antioxidant systems in ripening tomato fruits. Biol. Plantarum.

[29] Mondal K, Malhotra SP, Jain V, Singh R (2009). Oxidative stress and antioxidant systems in Guava (*Psidium guajava* L.) fruits during ripening. Physiol. Mol. Biol. Plants..

[30] García-Limones C (2002). Induction of an antioxidant enzyme system and other oxidative stress markers associated with compatible and incompatible interactions between chickpea (*Cicer arietinum* L.) and *Fusarium oxysporum* f. sp. *ciceris*. Physiol. Mol. Plant Pathol..

[31] Bradford MM (1976). A rapid and sensitive method for the quantitation of microgram quantities of protein utilizing the principle of protein-dye binding. Anal. Biochem..

[32] Surrey K (1964). Spectrophotometric Method for Determination of Lipoxidase Activity. Plant Physiol..

[33] Davis BJ (1964). Disc Electrophoresis -α Method And Application To Human Serum Proteins. Ann. N. Y. Acad. Sci..

[34] Mittler R, Zilinskas BA (1993). Detection of ascorbate peroxidase activity in native gels by inhibition of the ascorbate-dependent reduction of nitroblue tetrazolium. Anal. Biochem..

[35] Guikema JA, Sherman LA (1981). Electrophoretic profiles of cyanobacterial membrane polypeptides showing heme-dependent peroxidase activity. Biochim. Biophys. Acta..

[36] Clare DA (1984). Effects of molecular oxygen on detection of superoxide radical with nitroblue tetrazolium and on activity stains for catalase. Anal. Biochem..

[37] Beauchamp C, Fridovich I (1971). Superoxide dismutase: Improved assays and an assay applicable to acrylamide gels. Anal. Biochem..

[38] Ge Y (2017). Correction: Hydrogen sulfide alleviates postharvest ripening and senescence of banana by antagonizing the effect of ethylene. PLoS One..

