# Supplementary material for: Antioxidative capacity is highly associated with the storage property of tuberous roots in different sweetpotato cultivars
Source: Sci Rep. 2019 Jul 31;9:11141. doi: 10.1038/s41598-019-47604-8 (PMC6668466; doi:10.1038/s41598-019-47604-8)

Title page

**Antioxidative capacity is highly associated with the storage capacity of tuberous roots in different sweetpotato cultivars**

Jun Tang^1, †^, Si-Qi Wang^2, †^, Kang-Di Hu^2, †^, Zhong-Qin Huang^1^, Yan-Hong Li^2^, Zhuo Han^2^, Xiao-Yan Chen^2^, Lan-Ying Hu^2,3^, Gai-Fang Yao^2,*^, Hua Zhang^2,*^

^1^ *Xuzhou Institute of Agricultural Sciences of the Xuhuai District of Jiangsu Province, Xuzhou, Jiangsu 221131, PR China*

^2^ *School of Food and Biological Engineering, Hefei University of Technology, Hefei, Anhui 230009, PR China*

^3^*Anhui Province Key Laboratory of Functional Compound Seasoning, Anhui Qiangwang seasoning Food Co., Ltd., Jieshou 236500, PR* *China*

^†^ Si-Qi Wang, Jun Tang and Kang-Di Hu are co-first authors, and they have contributed equally to this work.

* Corresponding author: Tel.: +86 18919661103, Fax: +86-551-62901043, E-mail address: hzhanglab@hfut.edu.cn (H. Zhang); Tel.: +86 18256047576, E-mail address: yaogaifang@hfut.edu.cn (Gai-Fang Yao)

Table S1. The primers used in the quantitative PCR of gene expression assay.

| Primers | Sequence | Sequence ID |
| --- | --- | --- |
| *IbAPX* (forward) | 5’-CTCGACACTACCACTCCTATCT-3’ | itf09g09790 |
| *IbAPX* (reverse) | 5’-CGTGACTCCACCGACAAATAA-3’ |  |
| *IbSOD* (forward) | 5’-ACATCACGGTTGGAGAAGATG-3’ | itf13g19030 |
| *IbSOD* (reverse) | 5’-TCGGGATCACCATGAACAAC-3’ |  |
| *IbPOD* (forward) | 5’-GGCGGAGGATGCTGTTAAT-3’ | itf09g09800 |
| *IbPOD* (reverse) | 5’-GGGAAGGATGAGAAGGTTGAAG-3’ |  |
| *IbCAT3* (forward) | 5’-GTTCGGTTCTCCACTGTTATCC-3’ | itf07g00160 |
| *IbCAT3* (reverse) | 5’-CACCAGATCAAAGTTACCCTCTC-3’ |  |
| *IbLOX1* (forward) | 5’-TCTGTTGCTCAGTTCCTTCTC-3’ | itf15g12180 |
| *IbLOX1* (reverse) | 5’-CAGCTTGATCCCTCCTTCATAG-3’ |  |
| *IbTubulin* (forward) | 5’-CTCAAGAGGGTCTCAGCAATAC-3’ | itf04g29110 |
| *IbTubulin* (reverse) | 5’-TGTCAAGTAACGCCCATGTC-3’ |  |

Figure legends of supplementary figures:


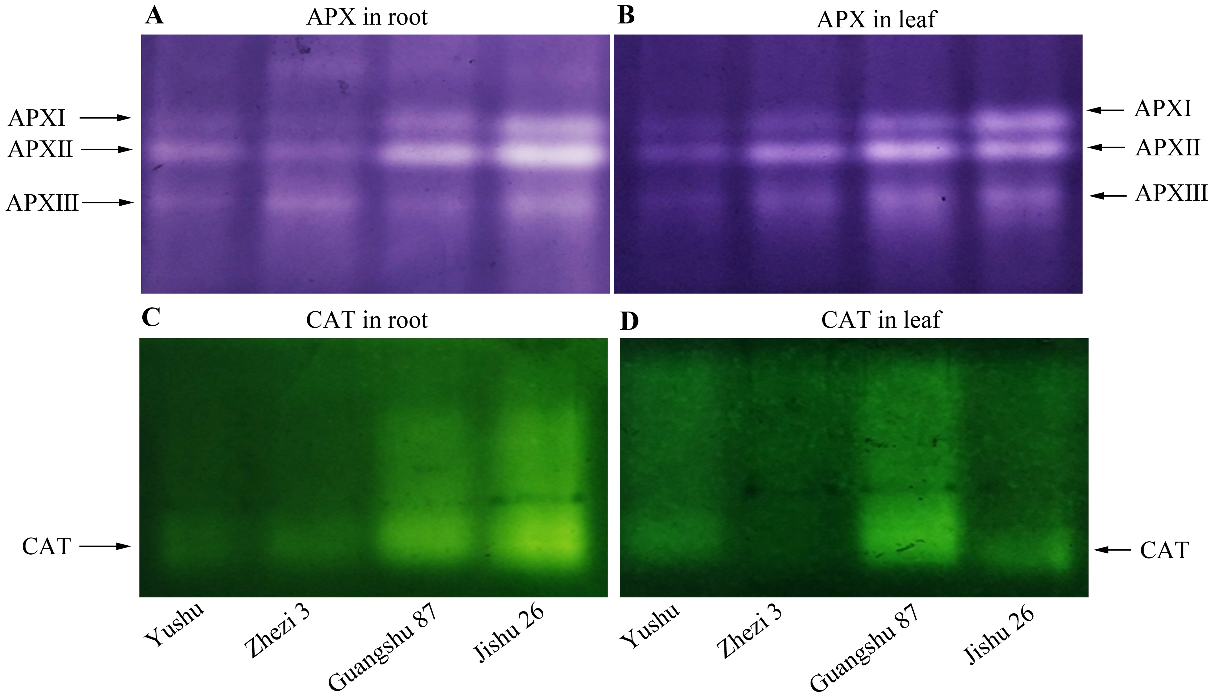


**Figure S1.** Native polyacrylamide gel electrophoresis (PAGE) of isozyme profile of ascorbate peroxidase (APX) and catalase (CAT) in the tuberous roots and leaves of sweet potato cultivars Yushu, Zhezi 3, Guangshu 87 and Jishu 26.


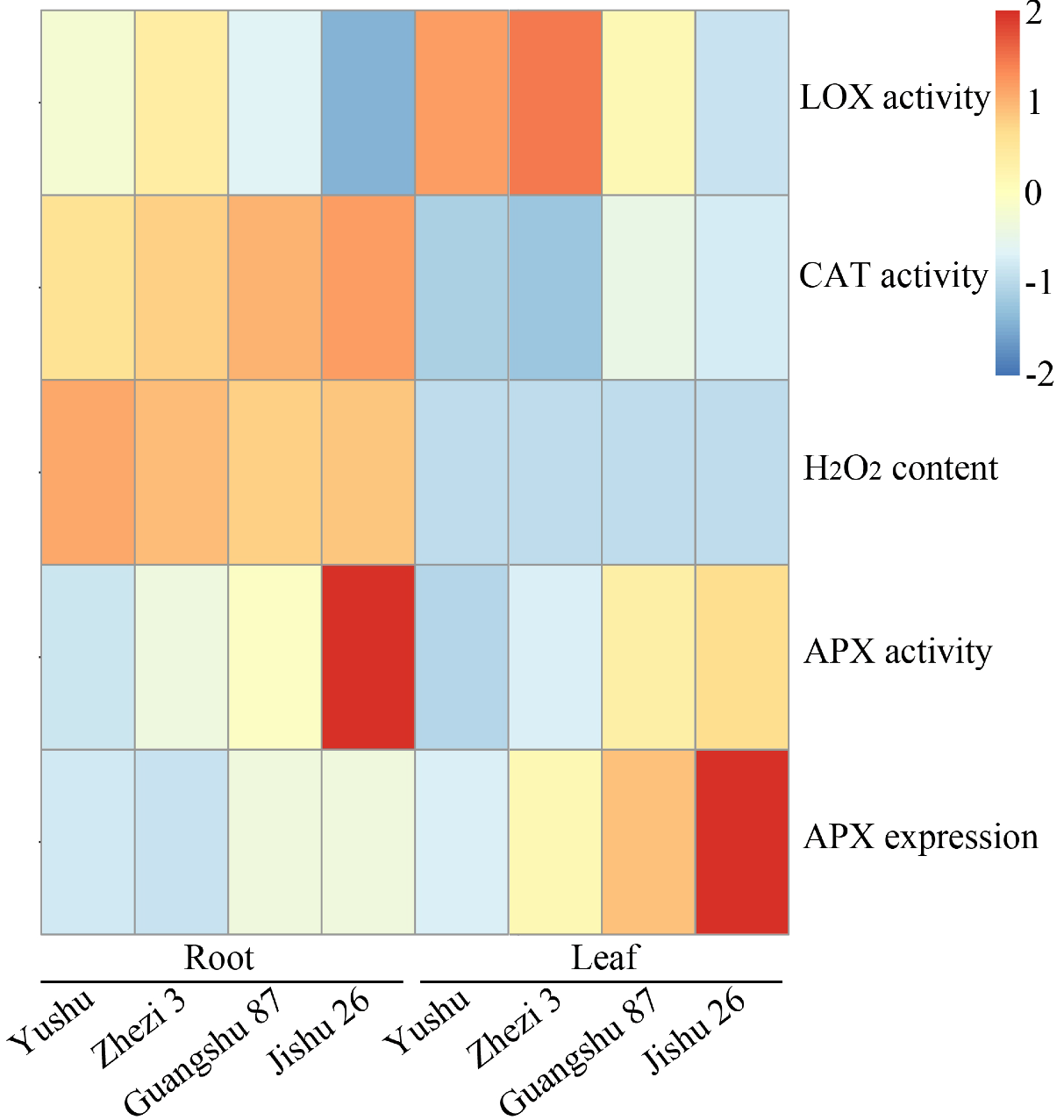


**Figure S2.** Heatmap based on the activities of lipoxygenase (LOX), catalase (CAT), ascorbate peroxidase (APX), the content of H_2_O_2_ and relative gene expression of itf09g09790 (*IbAPX*) in tuberous roots and leaves of sweet potato cultivars Yushu, Zhezi 3, Guangshu 87 and Jishu 26. Heat map scale bars are shown.


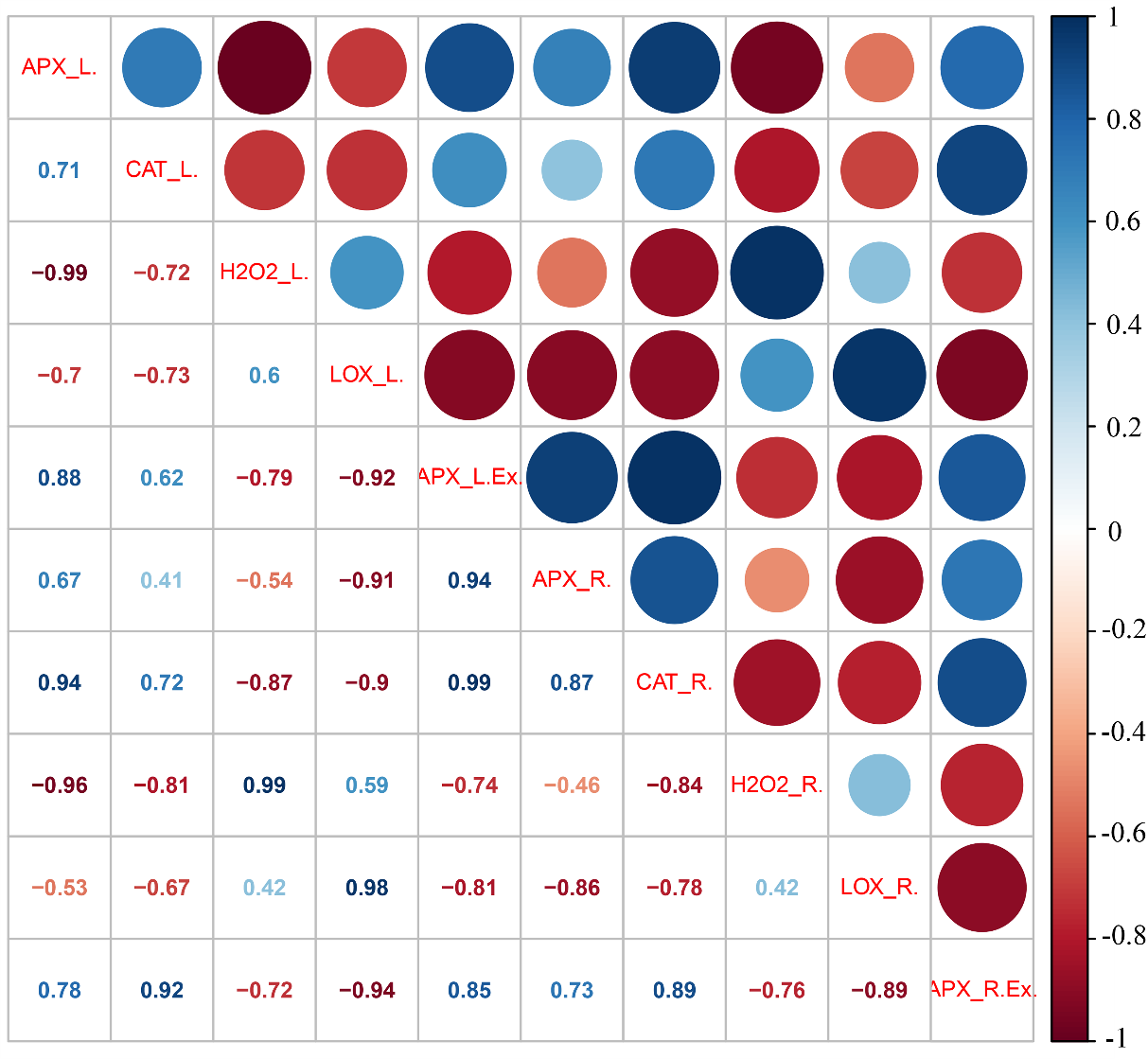


**Figure S3.** Correlation analysis among the parameters of ascorbate peroxidase (APX), catalase (CAT), lipoxygenase (LOX) and the data of hydrogen peroxide (H_2_O_2_) and gene expression of itf09g09790 (*IbAPX*) in the tuberous roots and leaves of sweet potato cultivars Yushu, Zhezi 3, Guangshu 87 and Jishu 26. Pearson’s correlation coefficient among data was analyzed using R scripts. R, abbreviation of root; L, abbreviation of leave; Ex, abbreviation of gene expression.


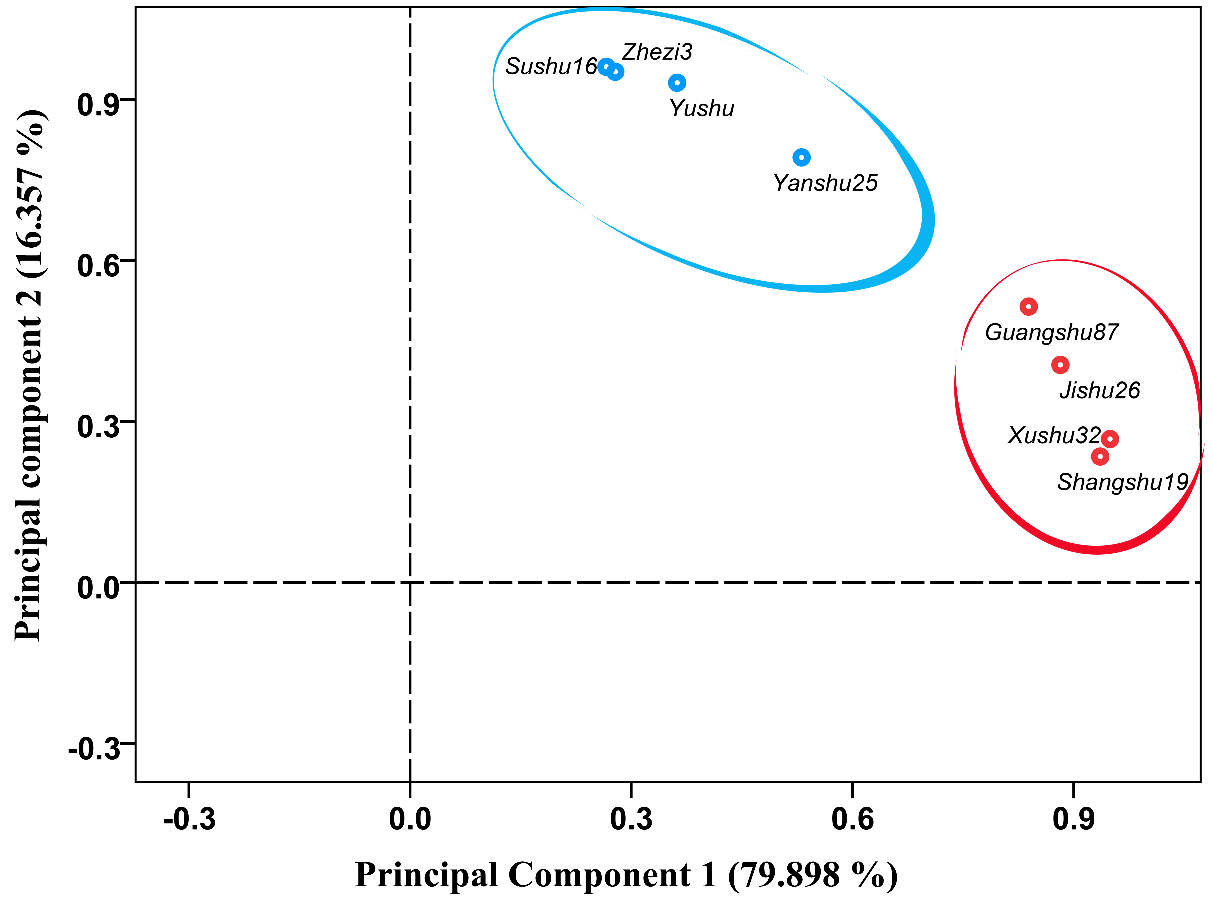


**Figure S4.** Principal component analysis to understand the relationship between cultivars of sweet potato and storage property.

Original picture of Fig. 1G and Fig. 3G


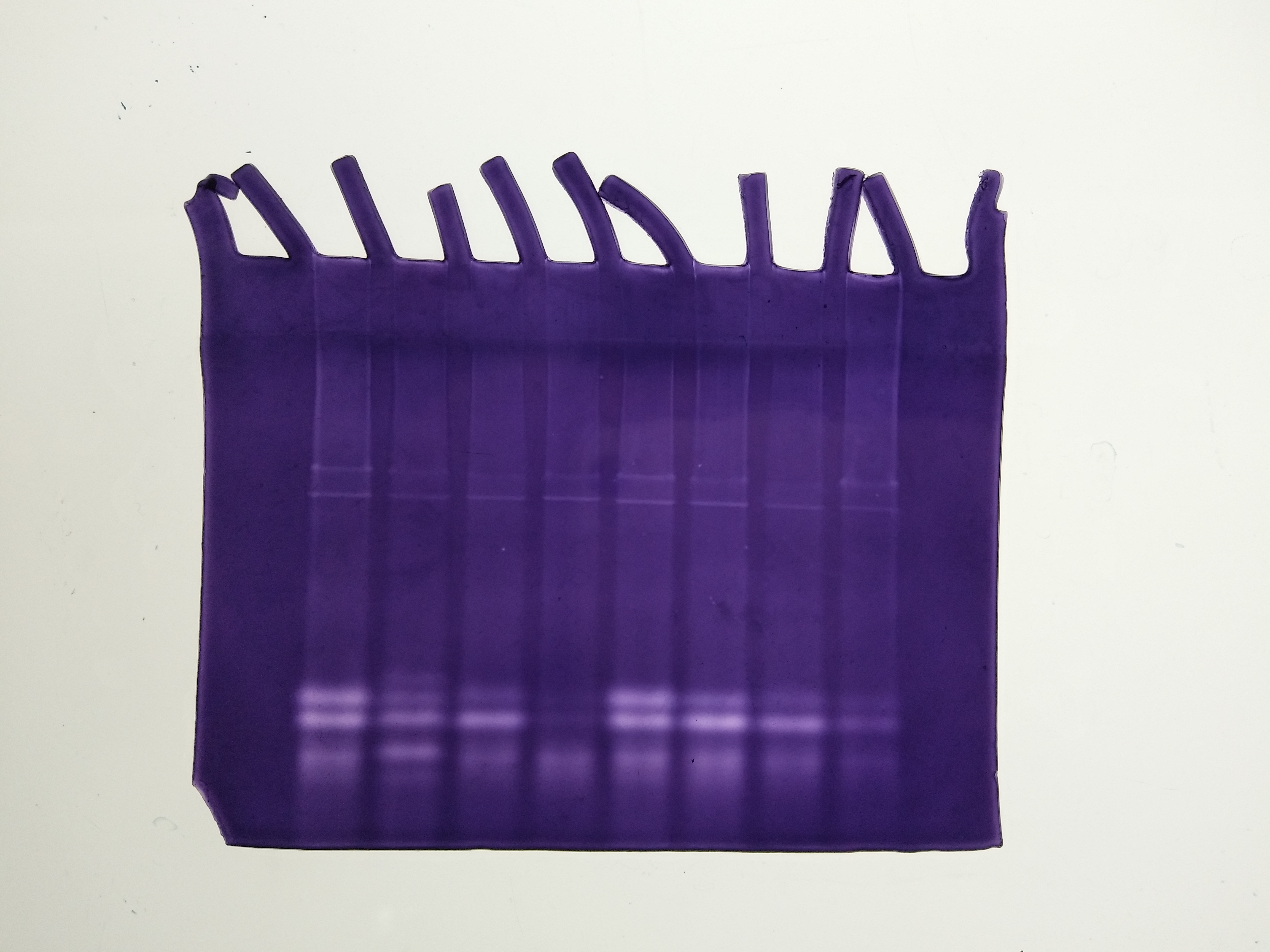


Original picture of Fig. 1H and Fig. 3H


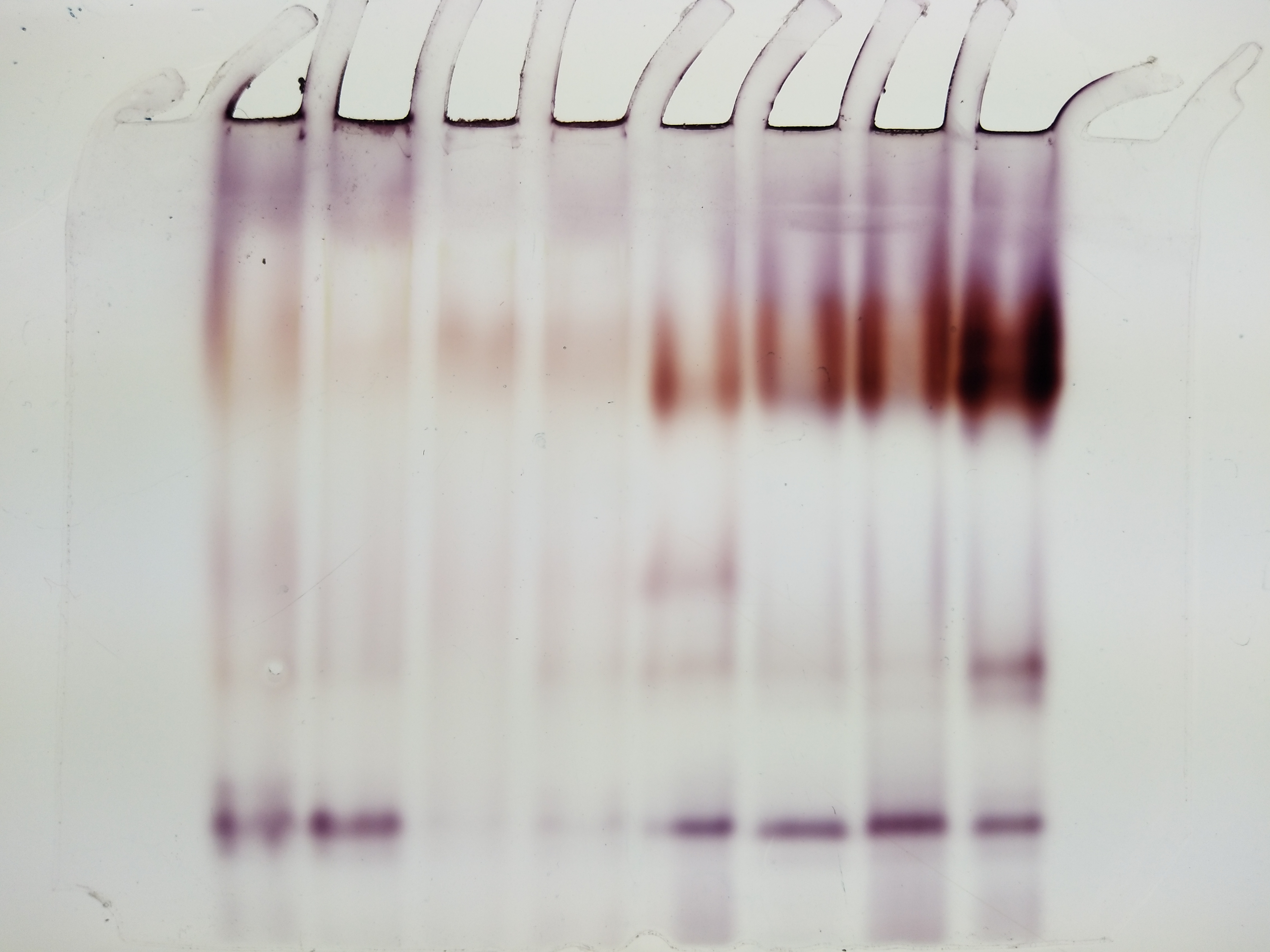


Original picture of Fig. 1I


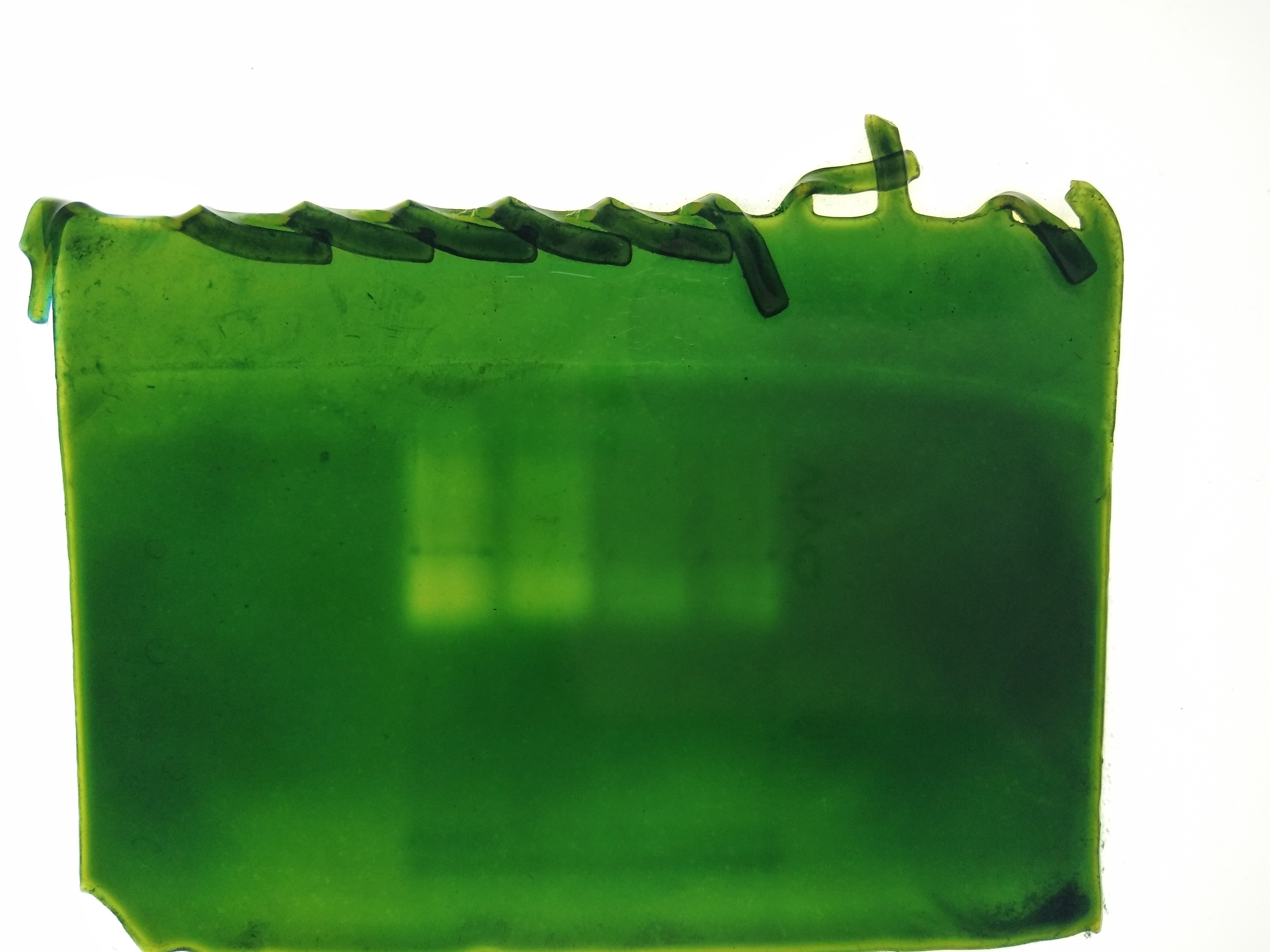


Original picture of Fig. 3I


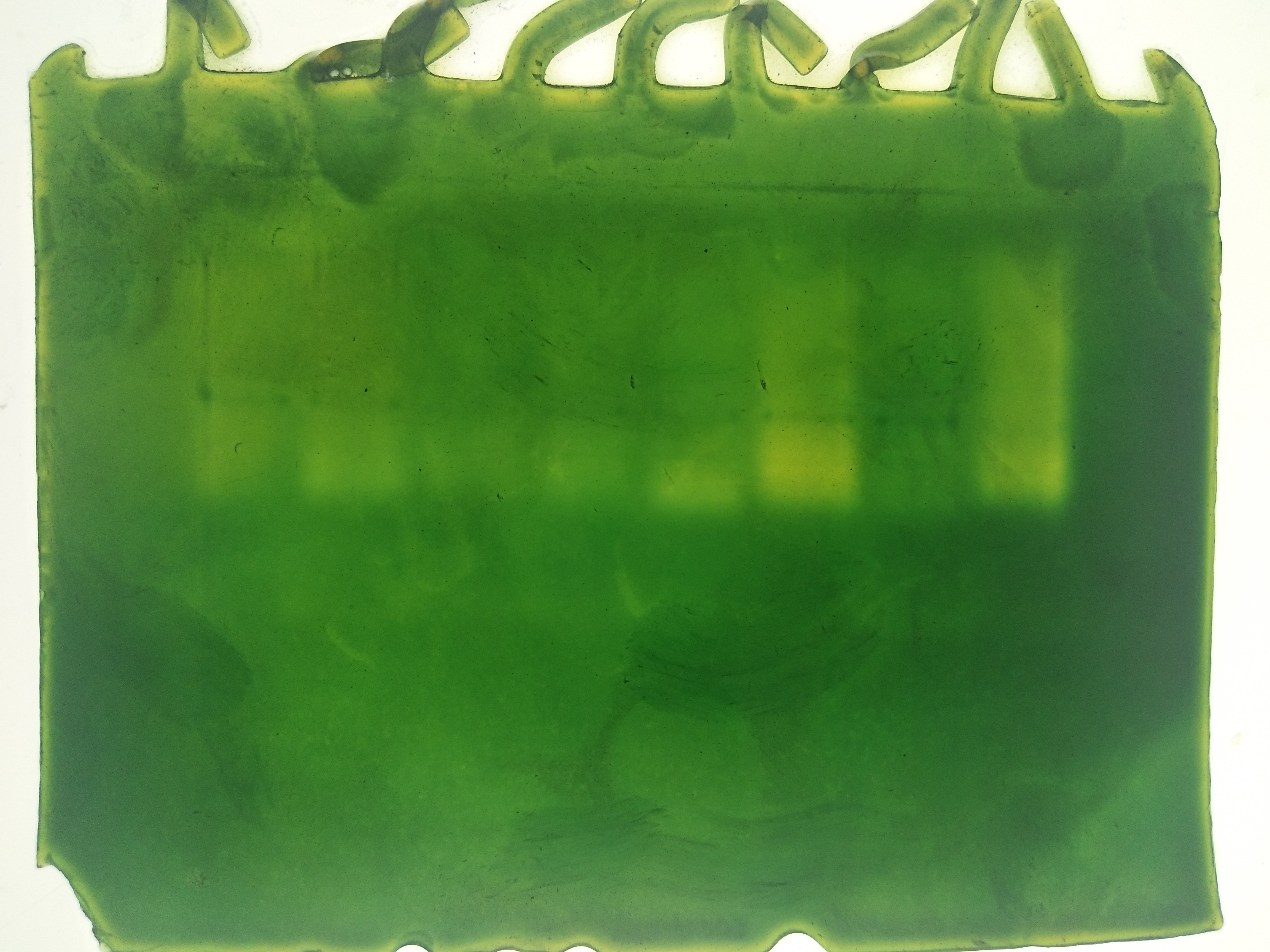


Original picture of Fig. 1J


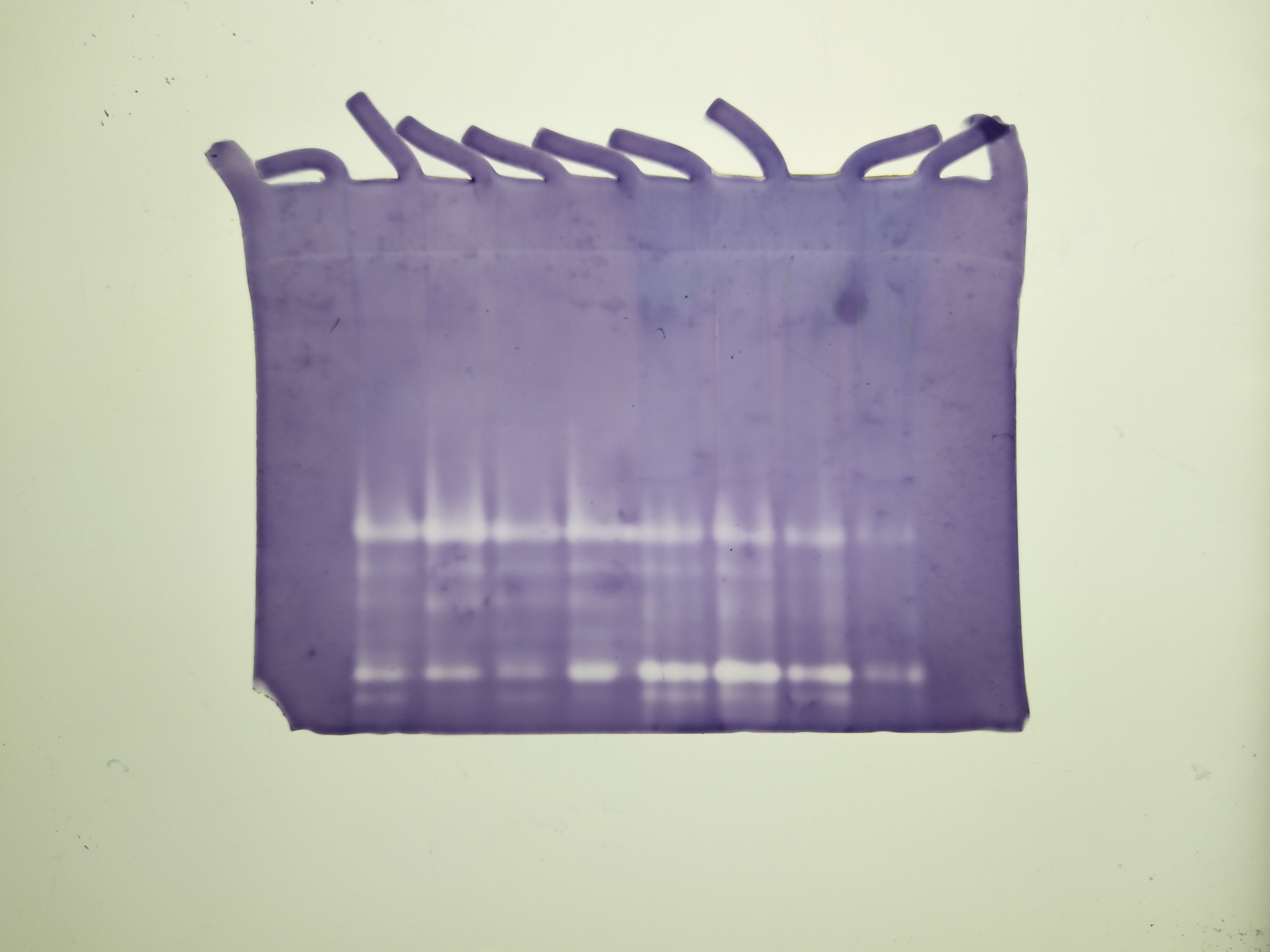


Original picture of Fig. 3J


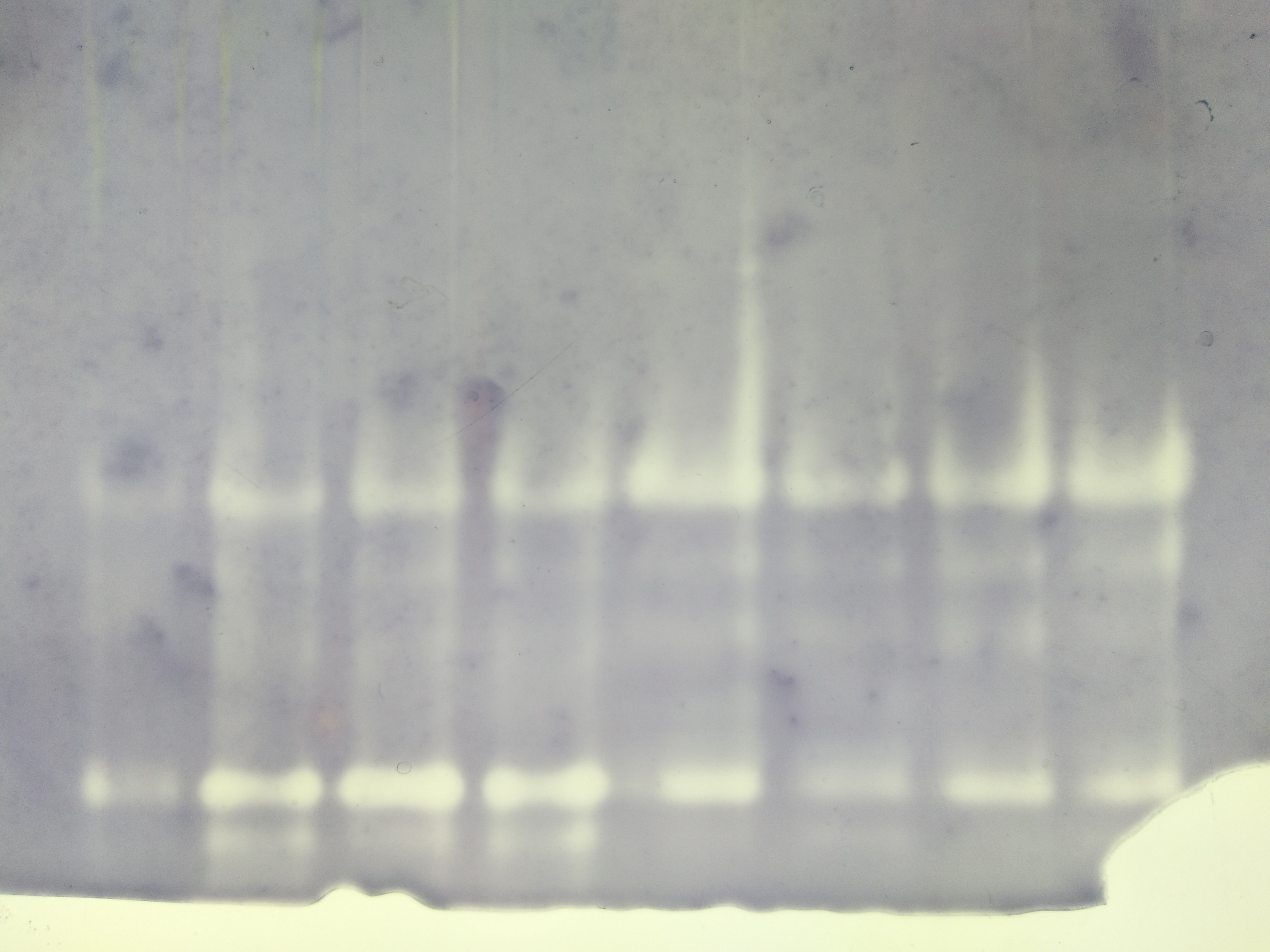

Supplement: Supplementary file 1 — Dataset 1 [file 41598_2019_47604_MOESM1_ESM.docx]
